# Comprehensive analysis of diagnosis and treatment in 99 cases of abdominal Schwannoma

**DOI:** 10.1002/cam4.70140

**Published:** 2024-08-19

**Authors:** Shaoqing Fan, Haiqian Wang, Xuemei Sun, Chunyue Gai, Ce Liang, Guiying Wang, Wenbo Niu

**Affiliations:** ^1^ Department of General Surgery The Fourth Hospital of Hebei Medical University Shijiazhuang Hebei China; ^2^ Department of Nursing The Fourth Hospital of Hebei Medical University Shijiazhuang Hebei China; ^3^ Department of Pathology The Fourth Hospital of Hebei Medical University Shijiazhuang Hebei China; ^4^ Department of Thoracic Surgery The Fourth Hospital of Hebei Medical University Shijiazhuang Hebei China; ^5^ Department of Pharmacology The Hebei Medical University Shijiazhuang Hebei China; ^6^ Department of General Surgery The Second Hospital of Hebei Medical University Shijiazhuang Hebei China

**Keywords:** abdominal schwannoma, gastrointestinal neuroectodermal tumor, GNET, S100, SOX10

## Abstract

**Purpose:**

Schwannoma is a rare mesenchymal tumor. In this study, we analyzed clinicopathologically 99 schwannomas.This retrospective study delves into the clinical, pathological, and immunohistochemical dimensions of abdominal schwannomas.

**Results:**

A cohort of 99 cases, comprising 4 malignant and 95 benign schwannomas, was meticulously examined. Clinical analysis revealed a notable gender distribution (1:1.7, male to female) and an average age of 58.5 years. The majority of cases were asymptomatic. A cohort of 99 cases, comprising 4 malignant and 95 benign schwannomas, was meticulously examined. Clinical analysis revealed a notable gender distribution (1:1.7, male to female) and an average age of 58.5 years. The majority of cases were asymptomatic. Tumor sizes ranged from 0.5 to 30 cm, with distinct locations in the stomach for most benign cases and the abdomen/small intestine for malignancies. Initial misdiagnoses were frequent. Pathological evaluations revealed distinct features, including Antoni A and B patterns, spindle cells, and lymphatic sheath structures in benign schwannomas. Malignant cases exhibited atypical cells, ulcers, and invasive growth. Immunohistochemical markers, such as S100, SOX10, and vimentin, consistently demonstrated positivity by contributing to accurate diagnoses. Treatment outcomes indicated a poor prognosis in malignant cases, with overall survival ranging from 10 to 41 months. Conversely, benign cases displayed no recurrence or metastasis during follow‐up, despite atypical behaviors.

**Conclusion:**

This study underscores the rarity of abdominal schwannomas and underscores the need for a comprehensive diagnostic morphology and immunohistochemistry. SOX10 emerges as a crucial and specific marker for accurate diagnosis. Further research is imperative to refine diagnostic protocols and enhance our understanding of the clinical behavior of abdominal schwannomas.

## Abstract 2

The study conducted a retrospective, large‐scale analysis of 99 cases of abdominal schwannomas. It unveiled the rare occurrence of malignant schwannomas in the small intestine and abdominal cavity, posing challenges in diagnosis and differential diagnosis. The presentation of novel histological and immunohistochemical features in the cases provides crucial insights for the accurate diagnosis of schwannomas. A detailed analysis of both benign and malignant cases establishes a foundation for future improvements in clinical management and treatment strategies.

## 1 INTRODUCTION

Schwannoma, a tumor arising from the nerve sheath of peripheral nerves, originates from neural crest cells and exhibits a microscopic structure reminiscent of clear cell sarcoma (CCS). Typically solitary with slow growth, it predominantly occurs in the intracranial and spinal cord regions, with rare instances in the gastrointestinal tract.^1^ Daimaru et al.^2^ identified a series of immunohistochemical records of 24 benign gastrointestinal schwannomas termed benign gastrointestinal neuroectodermal tumors (GNET). Gastrointestinal schwannomas, more prevalent in middle‐aged women,^3^ constitute 2%–6% of all mesenchymal tissue tumors.^4^ Malignant gastrointestinal schwannomas, exceptionally rare and commonly found in the small intestine, have sporadically been reported in the non‐intestinal abdominal cavity,^5^, ^6^ affecting diverse age groups. The lack of morphological specificity makes schwannomas prone to misdiagnosis as colon cancer and mesenchymal tumors. Early distant metastases and a dearth of specific treatments contribute to poor prognoses, necessitating further clinical and experimental research. This study retrospectively analyzes 99 abdominal schwannoma cases, encompassing 4 malignant cases (1 in the small intestine and 3 in the abdominal cavity) and 95 benign cases. The analysis includes scrutiny of clinical features, pathological characteristics, immunohistochemical profiles, treatment modalities, and survival prognoses.

## 2 PATIENTS AND METHODS

### 2.1 Case selection

A retrospective analysis was conducted on 99 cases of abdominal schwannomas diagnosed at the Fourth Hospital of Hebei Medical University between January 1, 2015, and June 1, 2023. Data retrieval included comprehensive information sourced from the Record Room, while the pathology department provided detailed pathological data. The dataset was found to be complete. Clinical parameters encompassed gender, age, medical history, clinical features, and the entire treatment trajectory.

Histological evaluations were carried out by two senior pathologists who meticulously reviewed HE‐stained sections, documenting gross specimen details, histological morphology, infiltration depth, local tissue expansion, as well as lymph node and distant metastasis occurrences. Immunohistochemical analyses involved a re‐evaluation of stained sections, including markers such as SOX100, CD34, CD117, DOG‐1, Ki67, Desmin, and Vimentin. Additionally, representative blocks were carefully selected for immunohistochemical staining analysis of SOX10 and GFAP.

This study received approval from the Ethics Committee of the Fourth Hospital of Hebei Medical University, and all patients provided informed consent before their inclusion in the study, adhering to ethical guidelines and standards.

### 2.2 Immunohistochemical staining

In accordance with the immunohistochemical staining protocol, representative sections underwent immunohistochemical staining utilizing the Benchmark ULTRA automatic staining instrument. Table 1 provides a comprehensive summary of the primary antibodies employed in this study. All immune markers were consistently stained for both positive and negative controls, ensuring methodological rigor and reliability.

**TABLE 1:** Primary antibodies used in the study.

Antibody	Source	Clone	Antibody dilution
SOX10	Maixin	EP268	1:100
GFAP	Maixin	MX047	1:100

## 3 RESULTS

### 3.1 Clinical features

#### 3.1.1 Basic feature

In the cohort of 99 cases, there were 37 males and 62 females, reflecting a gender distribution of 1:1.7 (male to female). The cases comprised 4 instances of malignant schwannoma (4%) and 95 cases of benign schwannoma (96%). The average age of the patients was 58.5 years, with a range from 19 to 82 years. Notably, 24.2% of patients were under 50 years old, while 75.8% were aged over 50 years. A detailed breakdown of clinical characteristics and pathological types is presented in Table 2.

**TABLE 2:** Clinical characteristics and pathological types.

Case No.	Sex	Age	Clinical symptoms and signs	Initial diagnosis	Endoscopic ultrasonography, EUS (Y or N)	Locations	Quantity	Comorbidities	Biological behavior	Size (cm)
1	Female	62	Abdominal distension and mass	Spindle cell liposarcoma	N	Abdominal cavity	2		Malignancy	30; 16
2	Male	19	Abdominal pain	Abdominal mesenchymoma	N	Abdominal cavity	1		Malignancy	16
3	Male	53	Abdominal distension, pain and intestinal obstruction	Malignant tumor of small intestine and multiple liver metastases	N	Small intestine	2		Malignancy	5; 4
4	Male	66	NA	Spindle cell liposarcoma	N	Abdominal cavity	1	Rectal cancer	Malignancy	4
5	Male	56	NA	GSTs	Y	Gastric antrum	1		Benign	3
6	Female	64	NA	Intraoperative findings	N	Transverse mesocolon	1	Gastric cancer	Benign	1.5
7	Female	53	Upper abdominal pain after hunger	GSTs	N	Gastric body	1		Benign	6
8	female	66	Abdominal distension	GSTs	Y	Gastric body	2		Benign	3.5; 1
9	Female	47	Abdominal distension	GSTs	Y	Gastric antrum	1		Benign	2.5
10	Male	62	NA	Intraoperative findings	N	Fundus of stomach	1	Esophagus cancer	Benign	1
11	Female	57	acid reflux	GSTs	N	Gastric body	1		Benign	6
12	Male	65	acid reflux	GSTs	Y	Gastric antrum	1		Benign	2
13	Male	54	NA	Intraoperative findings	N	Fundus of stomach	1	Esophagus cancer	Benign	0.5
14	Female	51	Upper abdominal pain	GSTs	Y	Gastric body	1		Benign	2.5
15	Male	63	Abdominal distension	GSTs	Y	Gastric antrum	1		Benign	8
16	Female	36	Upper abdominal pain	GSTs	Y	Gastric body	1		Benign	2.5
17	Female	61	Abdominal pain	GSTs	Y	Fundus of stomach	1		Benign	0.8
18	Female	48	Weak and diarrhea	GSTs	N	Gastric body	1		Benign	4
19	Male	66	NA	Exogenous tumor	Y	Esophagus	1		Benign	5.5
20	Male	46	NA	Intraoperative findings	N	Mesogaster	1	Gastric cancer	Benign	1
21	Female	45	NA	GSTs	Y	Gastric body	1		Benign	8
22	Female	70	Left abdominal pain	GSTs	N	Fundus of stomach	1		Benign	6
23	Female	54	NA	GSTs	N	Gastric body	1		Benign	5.5
24	Male	63	acid reflux	GSTs	Y	Gastric body	1		Benign	3
25	Male	47	Abdominal discomfort	Gastric fundus mass	Y	Fundus of stomach	1		Benign	3
26	Male	51	Hiccup	GSTs	Y	Gastric body	1		Benign	3.5
27	Female	64	NA	GSTs	Y	Gastric body	1		Benign	3
28	Female	50	NA	GSTs	Y	Gastric body	1		Benign	3
29	Female	48	NA	GSTs	Y	Gastric body	1		Benign	4
30	Female	77	NA	GSTs	Y	Gastric body	1		Benign	5.5
31	Female	67	NA	Ascending colon mass	N	Ascending colon	1		Benign	3
32	Male	62	Abdominal distension	Gastric fundus mass	N	Fundus of stomach	1		Benign	1
33	Female	55	Abdominal distension	GSTs or schwannoma?	Y	Gastric body	1		Benign	4
34	Female	61	Abdominal distension	Malignant tumor of small intestine	N	Small intestine	2	Mesenchymoma	Benign	5; 2.5
35	Male	60	NA	Intraoperative findings	N	Esophagus	2		benign	3
36	Male	66	NA	GSTs	N	Gastric body	1		Benign	6
37	Female	30	NA	GSTs	N	Gastric antrum	1		Benign	4
38	Male	82	NA	Schwannoma(pathology)	N	Ileocecal region	1		Benign	3.5
39	Male	71	Abdominal distension	GSTs	Y	Gastric body	1		Benign	1.5
40	Female	60	NA	GSTs	Y	Gastric body	1		Benign	4
41	Male	70	NA	GSTs	Y	Gastric antrum	1		Benign	4
42	Female	55	Left abdominal pain	GSTs	Y	Gastric antrum	1		Benign	3
43	Male	69	NA	Intraoperative findings	N	Esophagus and fundus of stomach	2		Benign	1.5
44	Female	47	NA	GSTs	Y	Gastric body	1		Benign	9
45	Male	67	NA	GSTs	Y	Gastric antrum	1		Benign	3.5
46	Female	73	NA	Intraoperative findings	N	Gastric body	1	Small intestine malignant fibroma	Benign	1.5
47	Female	73	acid reflux, diarrhea and belching	Gastric cancer	N	Gastric antrum	1		Benign	2.5
48	Female	67	NA	GSTs	Y	Gastric body	1		Benign	3
49	Female	50	NA	GSTs	N	Gastric antrum	1		Benign	5
50	Female	54	Abdominal pain	GSTs	N	Gastric body	1		Benign	3
51	Female	51	Abdominal discomfort	Schwannoma(CT)	Y	Gastric body	1		Benign	4
52	Male	61	NA	GSTs	Y	Fundus of stomach	1		Benign	2.5
53	Female	58	NA	GSTs	Y	Gastric body	1		Benign	6
54	Female	67	NA	Intraoperative findings	N	Fundus of stomach	1	Gastric cancer	Benign	3.5
55	Male	71	NA	GSTs	N	Gastric body	1		Benign	4.5
56	Female	36	Abdominal pain	Schwannoma(CT)	N	Gastric body	1		Benign	4.5
57	Female	57	NA	Ascending colon mass	Y	Ascending colon	1		Benign	2.5
58	Male	64	Abdominal distension and pain	GSTs	N	Gastric body	1		Benign	5.5
59	Female	75	NA	GSTs	Y	Gastric body	1		Benign	9
60	Female	57	NA	NET	N	Gastric antrum	1		Benign	2
61	Male	73	NA	GSTs	N	Gastric body	1	Descending colon cancer	Benign	4
62	Female	53	Belching	GSTs	N	Gastric antrum	1		Benign	6
63	Female	57	Abdominal distension, pain and acid reflux	GSTs or leiomyoma	Y	Gastric body	1		Benign	1.5
64	Female	66	NA	GSTs	N	Gastric body	1		Benign	5
65	Female	68	Decreased appetite	GSTs	Y	Gastric antrum	1		Benign	5
66	Female	63	Dysphagia	GSTs	N	gastric antrum	1		benign	8
67	Female	51	Abdominal discomfort	Leiomyoma	N	Gastric body	1		Benign	6
68	Female	72	Abdominal distension	Submucosal tumor	N	Ascending colon	1		Benign	3
69	Female	77	acid reflux	GSTs	Y	Gastric body	1		Benign	3
70	Male	35	Abdominal distension and pain	GSTs	N	Gastric antrum	1		Benign	2.5
71	Female	56	Abdominal distension	GSTs	Y	Gastric body	1		Benign	1.6
72	Male	71	NA	Intraoperative findings	N	Fundus of stomach	1	Esophagus cancer	Benign	2.5
73	Female	72	Abdominal distension	GSTs and liver metastasis	Y	Gastric body	1	Liver cancer	Benign	6
74	Male	67	NA	GSTs	N	Gastric body	1		Benign	1.2
75	Female	49	NA	GSTs	N	Gastric antrum	1		Benign	3
76	Female	49	Abdominal pain and melena	NET	N	Small intestine	1		Benign	4
77	Female	60	Abdominal discomfort and belching	GSTs	Y	Gastric body	1		Benign	3.5
78	Male	56	NA	GSTs	Y	Gastric body	1		Benign	1
79	Male	68	Abdominal discomfort and nausea	GSTs	Y	Gastric antrum	1		Benign	2.5
80	Male	69	NA	Intraoperative findings	N	Duodenum	1	Cholangiocarcinoma	benign	2
81	Female	64	NA	GSTs	N	Gastric antrum	1		Benign	8
82	Female	50	Abdominal distension and pain	GSTs	N	Gastric body	1		Benign	7
83	Female	63	NA	Submucosal mass	N	Small intestine	1		Benign	1
84	Female	63	Abdominal discomfort	GSTs	N	Duodenum	1		Benign	6
85	Female	72	acid reflux	GSTs	Y	Duodenum	1		Benign	0.8
86	Male	68	NA	Intraoperative findings	N	Fundus of stomach	1	Esophagus cancer	Benign	1.5
87	Female	65	NA	Intraoperative findings	N	Ascending colon	1		Benign	5.5
88	Female	53	acid reflux	GSTs	N	Gastric body	1		Benign	4
89	Male	59	NA	GSTs	N	Gastric body	1		Benign	3
90	Female	44	NA	GSTs	N	Ascending colon	1		Benign	1
91	Male	46	NA	GSTs	N	Gastric body	1		Benign	3.5
92	Male	67	NA	NET	Y	Rectum	1		Benign	1
93	Female	34	NA	GSTs	N	Gastric antrum	1		Benign	5
94	Male	58	NA	Intraoperative findings	N	Small intestine	1	Gastric cancer	Benign	0.6
95	Female	42	Abdominal distension and pain	GSTs	Y	Gastric antrum	1		Benign	2
96	Male	50	Abdominal distension	GSTs	Y	Gastric body	1		Benign	1.5
97	Female	59	NA	GSTs	Y	Gastric body	1		Benign	1
98	female	34	Abdominal discomfort	GSTs	Y	Gastric body	1		Benign	1.5
99	Female	71	NA	GSTs	Y	Sigmoid colon	1		Benign	2.5

### 3.2 Clinical features

Among the 99 patients diagnosed with schwannoma, 52.5% (52 cases) were asymptomatic. Notably, 39.4% of these asymptomatic cases were incidentally discovered during routine physical examinations. In 13.1% of patients, schwannoma was not initially identified preoperatively but was serendipitously detected during surgery while addressing other tumors. Common clinical symptoms encompassed abdominal distension (18.2%), abdominal pain (16.2%), acid reflux (8.1%), and abdominal discomfort (7.1%). Less frequently observed clinical symptoms included diarrhea (2%), intestinal obstruction (1%), abdominal mass (1%), belching (1%), decreased appetite (1%), dysphagia (1%), melena (1%), and weakness (1%).

#### 3.2.1 Initial diagnosis

**FIGURE 1:**
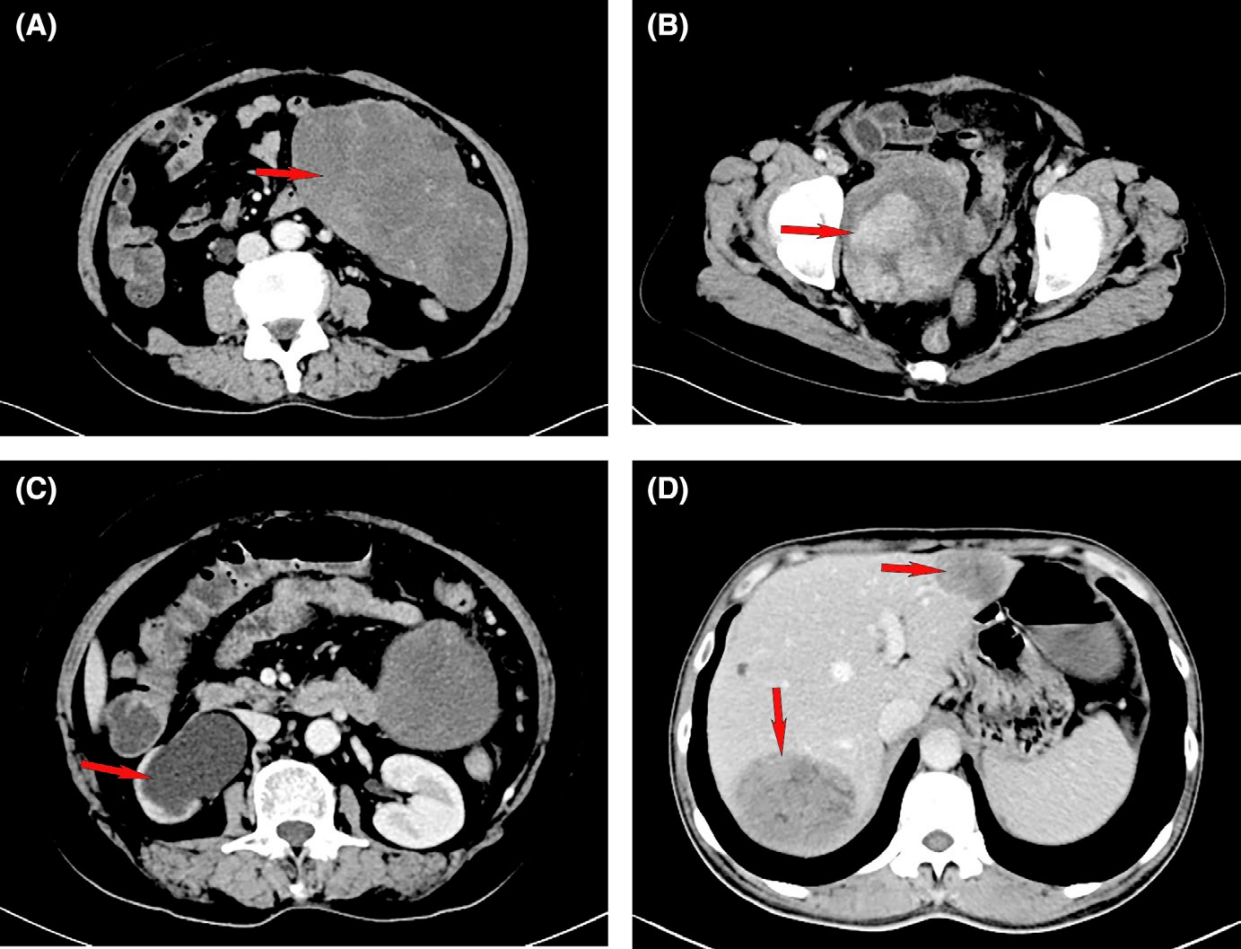
CT image characteristics. CT image from case 1 revealing an irregular mass (A) 25.0 cm × 15.0 cm and another (B) 15.0 cm × 15.0 cm in size located in the abdominal, with significant narrowing of the lumen and enhanced heterogeneity of mixed density (red arrow). (C) Left hydronephrosis and hydroureter. (D) CT image from case 3 revealing multiple liver metastases with the largest being 7.0 cm × 5.0 cm (red arrow).

Prior to surgery, only one patient received a definitive pathological diagnosis of schwannoma. Notably, this schwannoma was located in the ascending colon and exhibited growth beyond the intestinal mucosa. Among other cases, two were suspected to be schwannomas by CT physicians, while one case presented difficulty in distinguishing between GIST or schwannoma. A majority of cases, 67.7%, were initially classified as stromal tumors preoperatively, with 66 identified as gastrointestinal stromal tumors (GIST) and one as an abdominal stromal tumor. CT images are shown in Figure 1.

Additionally, three cases were initially considered as neuroendocrine tumors (NET), two as spindle cell liposarcoma, and two as adenocarcinomas of the small intestine. The remaining cases were not definitively characterized, being labeled as submucosal masses. In one instance, an FDG‐PET/CT examination revealed high metabolic activity, yet failed to provide a clear diagnosis or differentiation between benign and malignant conditions.

#### 3.2.2 Tumor size and location

The tumors exhibited a broad range of sizes, with the maximum diameter reaching 30 cm and the smallest measuring 0.5 cm. Categorically, 12 cases were ≤1 cm, 38 cases were >1 cm and ≤3 cm, 26 cases were >3 cm and ≤5 cm, and 23 cases were >5 cm. The average tumor size was 4.04 cm. Among the four cases of malignant schwannoma, three (75%) were located in the abdominal cavity, and one (25%) was in the small intestine.

Of the 95 cases identified as benign schwannoma, 78.9% were located in the gastric region, with 45 (47.4%) cases in the gastric body, 20 (21.1%) in the gastric antrum, and 10 (10.5%) in the fundus of the stomach. Other benign schwannomas were distributed across various locations, including five (5.3%) in the ascending colon, four (4.2%) in the small intestine, three (3.2%) in the duodenum, two (2.1%) in the esophagus, one (1.1%) in both the esophagus and fundus of the stomach, one (1.1%) in the sigmoid colon, one (1.1%) in the rectum, one (1.1%) in the ileocecal region, one (1.1%) in the mesogaster, and one (1.1%) in the transverse mesocolon.

### 3.3 Pathological features

**FIGURE 2:**
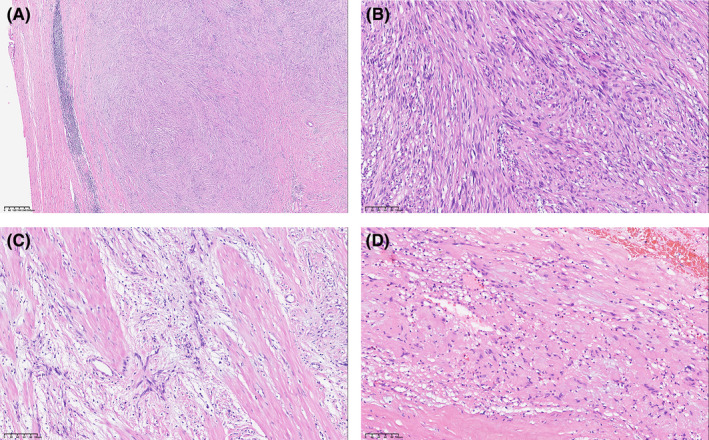
The histological characteristics of schwannoma. (A) Lymphatic sheath structure (5×). (B) Antoni A (20×). (C) Antoni B (20×). (D) Verocay body (20×).

The tumor cells predominantly occupy the muscularis propria layer, which is primarily composed of slender spindle cells featuring lymphatic sheath structures (Figure 2A). Two prevalent growth patterns, Antoni A (Figure 2B) and Antoni B (Figure 2C), are identified. Antoni A exhibits densely packed and incompletely arranged vortex‐shaped Verocay bodies (Figure 2D), while Antoni B histology showcases a group of relatively loose spindle cells. The cytoplasm is abundant and lightly stained with eosinophils, with varying amounts of visible collagen fibers interspersed between tumor cells.

**FIGURE 3:**
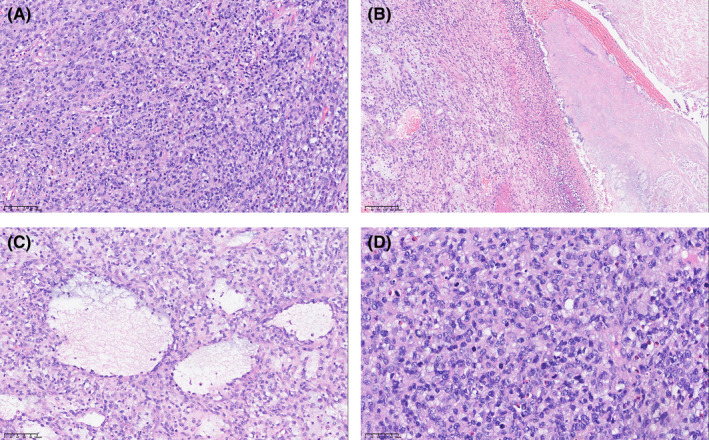
The histological characteristics of malignant schwannoma. (A) A large number of typical cells (20×). (B) Ulcer and invasive growth (20×). (C) cystic transformation (20×). (D) Higher mitotic rate (20×).

In one case of malignant schwannoma in the small intestine, atypical cells are observed (Figure 3A), accompanied by visible ulcers indicative of invasive growth (Figure 3B) and cystic transformation (Figure 3C). Notably, a higher mitotic rate (>60/10 HPF) is noted in these atypical cells (Figure 3D).

**FIGURE 4:**
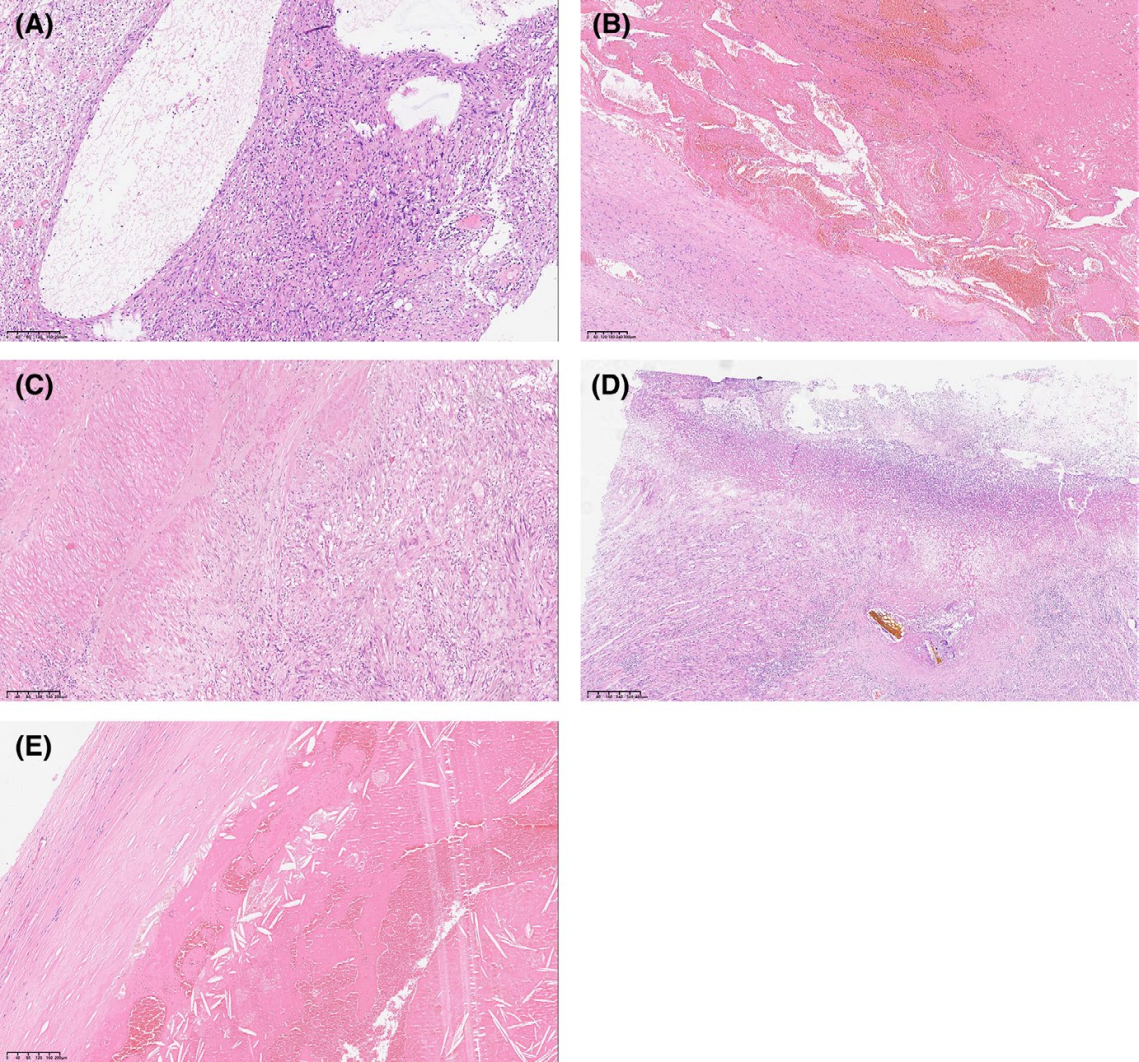
Rare biological behaviors in benign schwannoma. (A) Cystic transformation (10×). (B) Bleeding (5×). (C) Invasive growth (10×). (D) Ulcer (5×). (E) Necrosis (10×).

Benign abdominal schwannomas also display various biological behaviors, including cystic transformation (Figure 4A), bleeding (Figure 4B), invasive growth (Figure 4C), ulcers (Figure 4D), and necrosis (Figure 4E).

#### 3.3.1 Immunohistochemical results

**FIGURE 5:**
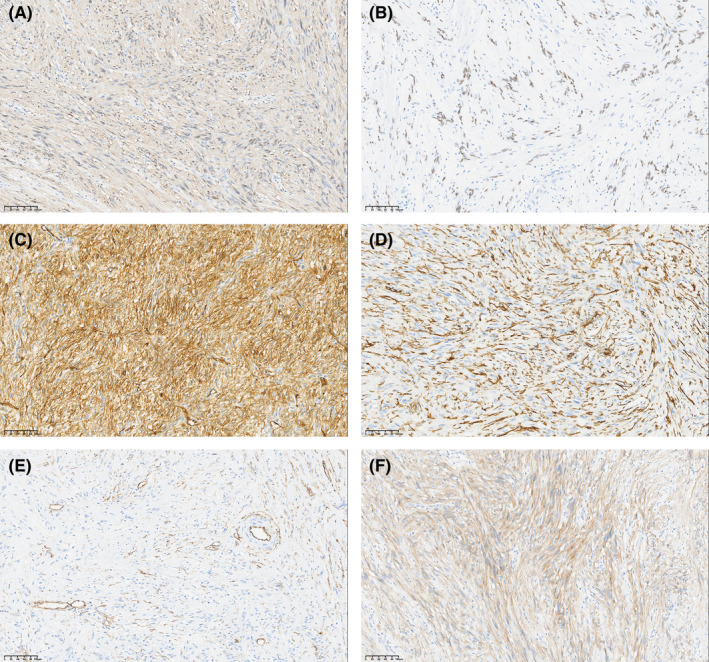
Immunohistochemical features (20×). (A) S100 expression displayed diffuse strong positivity in the nucleus and endochylema. (B) SOX10 only displayed strong positivity in the nucleus. CD34 displayed (C) strong positivity, (D) focal weak positivity, and (E) vascular positivity. (F) Negative results.

The immunohistochemical results are summarized in Table 3. S100 expression was observed in 98 cases (99.0%) and was characterized by diffuse nucleus and endochylema positivity (Figure 5A). SOX10 exhibited positive staining in 97 cases (98.0%), with strong positivity exclusively in the nucleus (Figure 5B). Vimentin demonstrated positive expression in 97 cases (98.0%). GFAP staining was positive in 68 cases (68.7%), with 27 cases (27.3%) displaying strong positivity, 25 cases (25.3%) exhibiting partial positivity, and 16 cases (16.2%) manifesting focal weak positivity. CD34 displayed vascular positivity in 54 cases (54.5%), with three cases (3.0%) showing strong positivity and three cases (3.0%) exhibiting focal weak positivity (Figure 5C–E). Desmin, CD117, and DOG‐1 predominantly showed negative results (Figure 5F). Desmin positivity was observed in 14 cases (14.1%), with one case (1.0%) displaying strong positivity, two cases (1.0%) showing weak positivity, and 11 cases (13.1%) manifesting focal weak positivity. CD117 demonstrated focal weak positivity in two cases (2.0%), and one case (1.0%) of DOG‐1 exhibited focal weak positivity.

**TABLE 3:** Immunohistochemical results.

Case No.	S100	GFAP	SOX10	CD34	Ki67	Des	Vim	CD117	DOG‐1
1	Patial+	(−)	(+)	Vessel+	(10%+)	Focal weak+	(+)	(−)	(−)
2	(+)	(−)	(−)	(+)	(50%+)	(−)	(+)	(−)	(−)
3	(+)	(−)	(+)	Vessel+	(40%+)	(−)	(+)	(−)	(−)
4	(+)	Focal weak+	(+)	Vessel+	(15%+)	(−)	(+)	(−)	(−)
5	(+)	(+)	(+)	Vessel+	(2%+)	(−)	(+)	(−)	(−)
6	(+)	(+)	(+)	Vessel+	(5%+)	(−)	(+)	(−)	(−)
7	(+)	(+)	(+)	(−)	(7%+)	(−)	(+)	(−)	(−)
8	(+)	(+)	(+)	(−)	(3%+)	(−)	(+)	(−)	(−)
9	(+)	(+)	(+)	(−)	(1%+)	(−)	(+)	(−)	(−)
10	(+)	Focal weak+	(+)	(−)	(1%+)	(−)	(+)	(−)	(−)
11	(+)	(+)	(+)	(−)	(2%+)	(−)	(+)	(−)	(−)
12	(+)	Patial+	(+)	Vessel+	(2%+)	(−)	(+)	(−)	(−)
13	(+)	Patial+	(+)	(−)	(5%+)	(−)	(+)	(−)	(−)
14	(+)	Patial+	(+)	(−)	(5%+)	(−)	(+)	(−)	(−)
15	(+)	Patial+	(+)	(−)	(12%+)	(−)	(+)	(−)	(−)
16	(+)	(+)	(+)	Vessel+	(3%+)	(−)	(+)	(−)	(−)
17	(+)	(+)	(+)	Vessel+	(3%+)	(−)	(+)	(−)	(−)
18	(+)	Patial+	(+)	Vessel+	(2%+)	(−)	(+)	(−)	(−)
19	(+)	Focal weak+	(+)	Vessel+	(3%+)	(−)	(+)	(−)	(−)
20	(+)	(−)	(+)	(−)	(2%+)	(−)	(+)	(−)	(−)
21	(+)	Patial+	(+)	Vessel+	(10%+)	(−)	(+)	(−)	(−)
22	(+)	Patial+	(+)	Vessel+	(5%+)	(−)	(+)	(−)	(−)
23	(+)	Focal weak+	(+)	Vessel+	(2%+)	Focal weak+	(+)	(−)	(−)
24	(+)	Focal weak+	(+)	Vessel+	(5%+)	(−)	(+)	(−)	(−)
25	(+)	Focal weak+	(+)	Vessel+	(8%+)	(−)	(−)	(−)	(−)
26	(+)	Patial+	(+)	(−)	(3%+)	(−)	(+)	(−)	(−)
27	(+)	(+)	(+)	(−)	(3%+)	(−)	(+)	(−)	(−)
28	(+)	(+)	(+)	Vessel+	(5%+)	(−)	(+)	(−)	(−)
29	(+)	(+)	(+)	(−)	(2%+)	(−)	(+)	(−)	(−)
30	(+)	(+)	(+)	Vessel+	(5%+)	(−)	(+)	(−)	(−)
31	(+)	(−)	(+)	(−)	(3%+)	(−)	(+)	(−)	(−)
32	(+)	(−)	(+)	Vessel+	(2%+)	(−)	(+)	(−)	(−)
33	(+)	Focal weak+	(+)	(−)	(5%+)	(−)	(+)	(−)	(−)
34	(+)	(−)	(+)	Vessel+	(1%+)	Focal weak+	(+)	(−)	(−)
35	(+)	(−)	(+)	Vessel+	(3%+)	Focal weak+	(+)	(−)	(−)
36	(+)	(−)	(+)	Vessel+	(5%+)	(−)	(+)	(−)	(−)
37	(+)	(+)	(+)	(−)	(2%+)	(−)	(+)	(−)	(−)
38	(+)	(−)	(+)	(−)	(8%+)	Focal weak+	(+)	(−)	(−)
39	(+)	Focal weak+	(+)	(−)	(3%+)	(−)	(+)	(−)	(−)
40	(+)	Patial+	(+)	Vessel+	(8%+)	(−)	(+)	(−)	(−)
41	(+)	Focal weak+	(+)	(−)	(3%+)	(+)	(+)	(−)	(−)
42	(+)	Patial+	(+)	Vessel+	(5%+)	(−)	(+)	(−)	(−)
43	(+)	(−)	(+)	Vessel+	(10%+)	Focal weak+	(+)	(−)	(−)
44	(+)	Focal weak+	(+)	Vessel+	(5%+)	(−)	(+)	(−)	(−)
45	(+)	(+)	(+)	Vessel+	(3%+)	(−)	(+)	(−)	Focal weak+
46	(+)	(+)	(+)	Vessel+	(8%+)	(−)	(+)	(−)	(−)
47	(+)	(−)	(+)	(+)	(<1%+)	(−)	(+)	Focal weak+	(−)
48	(+)	(+)	(+)	Vessel+	(2%+)	(−)	(+)	(−)	(−)
49	(+)	(−)	(+)	Vessel+	(3%+)	(−)	(+)	(−)	(−)
50	(+)	Patial+	(+)	(−)	(2%+)	(−)	(+)	(−)	(−)
51	(+)	(−)	(+)	Vessel+	(2%+)	(−)	(+)	(−)	(−)
52	(+)	(−)	(+)	Vessel+	(5%+)	(−)	(+)	(−)	(−)
53	(+)	(−)	(+)	Vessel+	(5%+)	(−)	(+)	(−)	(−)
54	(+)	(+)	(+)	(−)	(3%+)	(−)	(+)	(−)	(−)
55	(+)	(−)	(+)	Patial+	(<1%+)	(−)	(+)	(−)	(−)
56	(+)	Focal weak+	(+)	(−)	(8%+)	(−)	(+)	(−)	(−)
57	(+)	Focal weak+	(+)	(−)	(3%+)	(−)	(+)	(−)	(−)
58	(+)	(+)	(+)	(+)	(5%+)	(−)	(+)	(Weak+)	(−)
59	(+)	(+)	(+)	Vessel+	(3%+)	(Weak+)	(+)	(−)	(−)
60	(+)	(−)	(+)	Vessel+	(20%+)	Focal weak+	(+)	(−)	(−)
61	(+)	(+)	(+)	(−)	(3%+)	(−)	(+)	(−)	(−)
62	(+)	Patial+	(+)	Vessel+	(10%+)	Focal weak+	(+)	(−)	(−)
63	(+)	(−)	(+)	(−)	(3%+)	(−)	(+)	(−)	(−)
64	(+)	Patial+	(+)	Vessel+	(3%+)	(−)	(+)	(−)	(−)
65	(+)	Focal weak+	(+)	Vessel+	(8%+)	(−)	(+)	(−)	(−)
66	(+)	(+)	(+)	Vessel+	(5%+)	(−)	(+)	(−)	(−)
67	(+)	Patial+	(+)	Vessel+	(3%+)	(−)	(+)	(−)	(−)
68	(+)	(+)	(+)	Vessel+	(5%+)	(−)	(+)	(−)	(−)
69	(+)	Patial+	(+)	Vessel+	(5%+)	(−)	(+)	(−)	(−)
70	(+)	(+)	(+)	Vessel+	(2%+)	(−)	(+)	(−)	(−)
71	(+)	(+)	(+)	(−)	(1%+)	(−)	(+)	(−)	(−)
72	(+)	(−)	(+)	(−)	(2%+)	(−)	(+)	(−)	(−)
73	(+)	Patial+	(+)	Vessel+	(3%+)	(−)	(+)	(−)	(−)
74	(+)	Focal weak+	(+)	Vessel+	(1%+)	(−)	(+)	(−)	(−)
75	(+)	(−)	(+)	(−)	(1%+)	(−)	(+)	(−)	(−)
76	(+)	(−)	(−)	(−)	(10%+)	(−)	(+)	(−)	(−)
77	(+)	(+)	(+)	Vessel+	(2%+)	(−)	(+)	(−)	(−)
78	(+)	Patial+	(+)	Focal weak+	(3%+)	(−)	(+)	(−)	(−)
79	(+)	(+)	(+)	Vessel+	(2%+)	(−)	(+)	(−)	(−)
80	(+)	(−)	(+)	(−)	(3%+)	(−)	(+)	(−)	(−)
81	(+)	Patial+	(+)	Vessel+	(1%+)	(−)	(+)	(−)	(−)
82	(+)	Focal weak+	(+)	Vessel+	(3%+)	(−)	(+)	(−)	(−)
83	(+)	Focal weak+	(+)	Focal weak+	(8%+)	Focal weak+	(+)	(−)	(−)
84	(+)	(−)	(+)	(−)	(10%+)	(−)	(+)	(−)	(−)
85	(+)	(−)	(+)	Vessel+	(3%+)	(−)	(+)	(−)	(−)
86	(+)	(−)	(+)	(−)	(5%+)	(−)	(+)	(−)	(−)
87	(+)	Patial+	(+)	Vessel+	(5%+)	(−)	(+)	(−)	(−)
88	(+)	Patial+	(+)	Vessel+	(4%+)	(−)	(+)	(−)	(−)
89	(+)	Patial+	(+)	(−)	(5%+)	(−)	(+)	(−)	(−)
90	(+)	(−)	(+)	Vessel+	(1%+)	(−)	(+)	(−)	(−)
91	(+)	Patial+	(+)	(−)	(3%+)	(−)	(+)	(−)	(−)
92	(−)	(−)	(Weak+)	(−)	(2%+)	(−)	(+)	(−)	(−)
93	(+)	(−)	(+)	(−)	(3%+)	(−)	(+)	(−)	(−)
94	(+)	(−)	(+)	(−)	(2%+)	(−)	(+)	(−)	(−)
95	(+)	Patial+	(+)	(−)	(3%+)	Focal weak+	(+)	(−)	(−)
96	(+)	(+)	(+)	Vessel+	(3%+)	(−)	(−)	(−)	(−)
97	(+)	Patial+	(+)	Vessel+	(8%+)	Focal weak+	(+)	(−)	(−)
98	(+)	Patial+	(+)	(−)	(1%+)	(Weak+)	(+)	(−)	(−)
99	(+)	(−)	(+)	(−)	(<1%+)	(−)	(+)	(−)	(−)

#### 3.3.2 Treatment results and follow‐up

As of March 1, 2024, five individuals were lost to follow‐up. Among the four patients diagnosed with malignant schwannoma who underwent surgical treatment, postoperative pathology revealed no relevant regional lymph node metastasis. Unfortunately, all four patients have since succumbed to the disease. Patient 1 declined detailed follow‐up, resulting in an overall survival (OS) of 10 months. Patient 2, a 19‐year‐old at the time of onset, was discovered to have multiple bone metastases in the pelvis bone 18 months post‐surgery due to hip and thigh pain. The patient opted out of further treatment, indicating a progression‐free survival (PFS) of 18 months and an OS of 21 months. Patient 3 initially presented with small intestinal schwannoma accompanied by intestinal obstruction and liver metastasis, experiencing a PFS of 12 months. Following surgery, the patient opted for oral Chinese herbal medicine and achieved an OS of 18 months. Notably, no adjuvant chemotherapy or radiotherapy was administered in these cases. Patient 4, diagnosed with concurrent rectal cancer, underwent 6 cycles of CapeOx chemotherapy and 25 doses of 50.4Gy radiotherapy post‐surgery, resulting in a PFS of 35 months. Subsequently, the patient developed liver and lung metastases and discontinued relevant treatment, with an OS of 41 months. Due to the lack of post‐metastasis pathological examination, the source of metastasis could not be conclusively determined as either pelvic malignant schwannoma or rectal cancer.

The remaining 90 patients with benign schwannoma did not experience recurrence or metastasis during the follow‐up period, underscoring the relatively favorable prognosis associated with this category of abdominal schwannomas.

## 4 DISCUSSION

Abdominal malignant schwannoma is a rare entity, and the existing literature primarily consists of case reports or small series studies. This retrospective study presents a comprehensive analysis of a substantial number of cases encompassing both benign and malignant schwannomas. The primary objective is to elucidate the clinical and pathological features of schwannomas, seeking to identify more sensitive immunohistochemical indicators. By systematically examining a larger dataset, this study aims to contribute valuable insights into the characteristics of abdominal schwannomas, paving the way for a more nuanced understanding of this rare pathology.

Schwannomas predominantly occur in the central nervous system or are associated with spinal nerve roots,^7^ commonly presenting in the head and neck, upper and lower extremities, posterior mediastinum, and retroperitoneum. There have even been studies reporting exceedingly rare cases of hepatic schwannomas.^8^ Hanh Thi Dau et al.^9^ discovered that pelvic schwannomas originate from the sacral nerves or the peripheral nerve plexus of the lower abdomen. Gastrointestinal Neuroectodermal Tumors (GNET) initially characterized by Zambrano and displaying characteristics akin to Clear Cell Sarcoma (CCS).^10^ This study reveals a rarity of GNET as a submucosal tumor, more prevalent in the gastric tract than the intestine. Among the cases analyzed, 17 occurred in the duodenum, small intestine, and colon, while 75 occurred in the stomach, resulting in a gastric‐to‐intestinal ratio of 1:4.35. Interestingly, malignant schwannomas are more frequently found in the abdominal cavity and small intestine than in the stomach.

Most schwannomas originated from the muscularis propria layer, with 35 cases growing outside the gastric wall and 40 cases growing inside. The average diameter was 4.04 cm, and clinical manifestations lacked specificity, often discovered incidentally during routine examinations or surgery. Preoperative pathology presented challenges in diagnosis, with only one case initially identified as a schwannoma. Large tumors could lead to abdominal mass and intestinal obstruction, mimicking features of gastrointestinal stromal tumors (GIST).CT scans depicted schwannomas as circular soft tissue masses with clear boundaries, sometimes causing intestinal narrowing, challenging the distinction from GIST. Malignant cases presented with ulcers and infiltrative growth, posing challenges in distinguishing from abdominal CCS. Endoscopic manifestations included gastrointestinal tract narrowing and submucosal tumors, occasionally leading to ulcerative masses, potentially misdiagnosed as colon cancer or stromal tumors. Mucosal biopsy yielded limited information, often with a low positive rate, complicating the differentiation from other mesenchymal tumors. Endoscopic ultrasound examination, while useful in some cases, had limitations in diagnosing schwannomas, as they typically appeared in the fourth layer as low‐echogenicity masses originating from the mucosal layer.^11^ The echoes exhibited uniform reduction, and low echogenicity halos were visible at the mass edges, complicating differentiation from stromal tumors.^12^ The study underscores the complexity and challenges in diagnosing abdominal schwannomas, emphasizing the need for comprehensive diagnostic approaches.

Gross specimens of schwannomas typically exhibit a round shape with clear boundaries, a complete capsule, and a grayish‐yellow cross‐section. Notably, cystic transformation, necrosis, bleeding, invasive growth, and calcification are uncommon findings in conventional schwannomas.^13^ However, in this study, these atypical features were identified, yet the postoperative follow‐up revealed a favorable prognosis without any malignant behaviors like recurrence or metastasis, which are characteristics also observed in malignant schwannomas. Microscopically, the histopathological features of schwannomas are primarily characterized by slender bipolar spindle‐shaped cells with low nuclear pigmentation, a low mitotic rate, and variable cell morphology. Occasionally, a peripheral sleeve‐like lymphocyte infiltration, including a lymphatic sheath structure, benign nuclear dysplasia, and positive S‐100 protein immunostaining, is observed, serving as distinctive features of gastrointestinal schwannomas. This distinction sets schwannomas apart from other spindle cells of smooth muscle origin, such as stromal tumors, fibromas, or leiomyomas.^2^ Scholars like Ueyama contend that most Gastrointestinal Stromal Tumors (GIST) exhibit smooth muscle differentiation, thereby differentiating them from schwannomas.^14^

Named after Nils Ragnar Eugene Antoni, a Swedish neuropathologist, the Antoni A and Antoni B classifications represent the two predominant growth forms of schwannomas. Antoni A histology reveals a bundle‐like shape with slender, spindle‐shaped cells forming a palisade‐like or incomplete vortex‐like arrangement, often featuring Verocay bodies. Antoni B histology showcases a group of relatively loose spindle‐shaped cells within a myoid stroma.^15^ Interestingly, in this study, gastric schwannomas lacked verocay bodies, Antoni B areas, and thick‐walled blood vessels, aligning with existing literature suggesting these distinctions between gastric and peripheral schwannomas.^16^ This thorough examination of histopathological features contributes to a more nuanced understanding of the unique characteristics and variations observed in abdominal schwannomas.

The pathological diagnosis of schwannoma crucially relies on immunohistochemical results. In this study, tumor cells exhibited diffuse strong positivity in 98 cases (99%) for S100, 97 cases (98%) for SOX10, and 97 cases (98%) for Vimentin. The term S100 was initially introduced in 1965,^17^ and subsequent research has indicated its high expression in neuroma, neurofibroma, granular cell tumor, and all Schwann cell‐derived nerve sheaths.^18^ SOX10, a neural crest transcription factor, plays a pivotal role in the regulation, maturation, and maintenance of Schwann cells and melanocytes. In 2008, Nonaka et al.^19^ discovered, through immunohistochemistry, a 100% positive rate of SOX10 expression in schwannomas and a 97% positive rate in melanomas. While S100 is significantly expressed in meningiomas and fibromas, SOX10 exhibits expression exclusively in tumors derived from schwannomas and melanocytes. S100, though highly sensitive (97%) in distinguishing schwannomas, lacks specificity, with SOX10 emerging as the most sensitive and specific biomarker.^20^ However, it is essential to note that, fundamentally, HE staining morphology holds diagnostic significance, encompassing features such as Antoni growth patterns, Verocay bodies, lipid‐laden cells, and thick‐walled hyalinized vasculature.^21^

CD34 is a sialylated transmembrane glycoprotein originally described as a hematopoietic progenitor cell antigen in myeloid stem cells. Additionally, Miettinen et al. propose that CD34 is expressed in certain fibroblast‐like or non‐committed mesenchymal cells, including dermal fibroblasts, perivascular mesenchymal cells, and some stromal tissues, implying their nature as primitive, uncommitted mesenchymal cells possibly related to stromal fibroblasts.^22^ In 1998, Hirota and colleagues discovered that gastrointestinal stromal tumors (GISTs) originating from interstitial cells of Cajal harbor c‐KIT mutations and express KIT (CD117).^23^ KIT is a gene‐encoding receptor tyrosine kinase protein that is activated in GIST tumors. Tumors outside of GISTs occurring in the gastrointestinal tract typically test negative for KIT and have never been expressed in gastric schwannomas.^24^ Moreover, the absence of CD34 or c‐kit protein expression can aid in distinguishing schwannoma from gastrointestinal stromal tumors (GIST).^25^ However, in this study, CD34 exhibited strong positivity in 3 cases (3.0%), with one case identified as malignant schwannoma in the abdominal cavity and two cases located in the stomach. Three cases demonstrated weak positivity (3.0%), with one in the small intestine and two in the stomach. Studies have indicated that CD34 can label fibroblast‐like cells in the neurointima of normal neural tissue, and CD34‐positive cells are present in all peripheral nerve sheath tumors.^26^ In classical schwannoma and its variants, there is a notable discrepancy in the reported positive rate of CD34. Some spindle or star‐shaped cells in the tumor capsule and Antoni B region exhibit CD34 positivity and S‐100 protein negativity, with the opposite expression pattern in the Antoni A region. In pigmented schwannoma, the Antoni A and Antoni B regions may not be clearly delineated. In most cases, tumor cells are CD34‐negative, with only two reports noting diffuse strong positivity for CD34.^27^

GFAP positivity serves as a valuable marker in identifying components of glioblastoma, with the positive rate and depth of cell staining being linked to the differentiation of astrocytomas.^28^ In this study, 68 cases (68.7%) exhibited positive staining for GFAP. It is widely acknowledged that peripheral nerves are more prone to diffuse GFAP expression than cranial nerve‐associated schwannomas.^19^ Early observations of schwannomas suggest that GFAP positivity may be more prevalent in peripheral spinal, retroperitoneal, and mediastinal schwannomas.^29^ Some studies propose that the expression of keratin in schwannomas is attributed to cross‐reactivity with GFAP, as both keratin and GFAP are partially homologous proteins. The cross‐reactivity of AE1/AE3 with other intermediate filament proteins, such as GFAP, observed in brain and glioma tissues could explain the widespread presence of keratin and GFAP positivity in specific retroperitoneal schwannomas.^30^

Due to the predominantly benign nature of gastrointestinal schwannomas, the overall prognosis for patients is generally favorable. Although the incidence of malignant schwannoma is low, its highly invasive and rapid progression poses a significant challenge. The overall survival (OS) of the four malignant cases in this study ranged from 10 to 41 months. Bohlok et al.^4^ reported that, in their study of colorectal schwannomas, 3 out of 95 cases (3.1%) were malignant, with factors such as mitotic rates >5 per high‐power field (HPF), Ki‐67 index >10%, and tumor volume greater than 5 cm considered indicative of malignancy. Long‐term follow‐up to assess local recurrence and distant metastasis plays a crucial role in confirming the malignant nature of schwannomas, although this study's follow‐up parameters did not align with that methodology.

Notably, all benign cases in this study did not experience recurrence or metastasis during follow‐up, even in instances where there were observed biological behaviors such as ulcers, bleeding, and necrosis. This suggests that, while these behaviors may raise suspicion, the primary differentiation between benign and malignant tumors still heavily relies on the morphological characteristics observed through hematoxylin and eosin (HE) staining. Given the rarity of this tumor, the expertise of pathologists becomes paramount in accurately differentiating and diagnosing benign and malignant cases. The study findings indicate that malignant tumors typically exhibit features such as abundant, dense tumor cells of varying sizes, deep nuclear staining, significant atypia, increased mitotic figures, and evident biological behaviors like ulcers, invasive growth, bleeding, and necrosis. This comprehensive understanding underscores the importance of both morphological assessment and long‐term follow‐up in establishing accurate diagnoses and prognostic evaluations for gastrointestinal schwannomas.

The primary treatment for schwannoma is reliant on complete surgical resection, with an emphasis on achieving a sufficient margin distance as the optimal approach. Tumor recurrence is typically associated with incomplete resection and insufficient margins. Although it's biological behavior shares similarities with sarcomas, peripheral lymph node metastasis is rare, and radical surgical resection is not always necessary. For tumors located in the submucosal layer, treatment options may include endoscopic resection or transanal resection. In the case of general benign schwannomas, adjuvant treatment is not recommended post‐surgery. However, the understanding of malignant schwannomas remains limited, and there is currently no consensus on the optimal scope of surgical resection and subsequent treatment for malignant tumors.^31^ Malignant schwannomas often exhibit a propensity for liver, lung, bone, and other metastases, resulting in a very poor prognosis. While the common chemotherapy protocol includes ifosfamide and doxorubicin, the efficacy is often unsatisfactory. Some studies suggest that malignant schwannomas may respond favorably to a combination therapy involving the tyrosine kinase inhibitors Crizotinib and Pazopanib.^32^

Given the rarity of schwannomas and the challenges associated with preoperative diagnosis, a comprehensive review of the clinical characteristics of this disease proves valuable for clinicians encountering abdominal schwannomas. This understanding can contribute to improved decision‐making regarding treatment strategies and postoperative management.

## 5 CONCLUSION

In summary, abdominal schwannoma is a rare tumor characterized by spindle cells and exhibits a notably low malignant incidence rate. Malignant cases demonstrate highly invasive biological behavior, frequently leading to short‐term recurrence and hematogenous metastasis, either at the initial diagnosis or shortly after surgery. Immunohistochemical staining, particularly utilizing tumor markers such as S100 and SOX10, holds decisive significance in accurately diagnosing schwannoma. The limitations inherent in retrospective research underscore the need for ample follow‐up data, clinical trials, and additional molecular studies to enhance our understanding of these rare malignant tumors. Further exploration in these areas is imperative for advancing the knowledge and management of abdominal schwannomas.

## AUTHOR CONTRIBUTIONS

**Wenbo Niu:** Conceptualization (lead); writing – review and editing (lead). **Shaoqing Fan:** Conceptualization (lead); writing – original draft (equal). **Haiqian Wang:** Investigation (lead); project administration (equal); writing – original draft (equal). **Xuemei Sun:** Data curation (equal); formal analysis (equal); writing – original draft (equal). **Chunyue Gai:** Data curation (equal); software (equal); writing – original draft (equal). **Ce Liang:** Data curation (equal); writing – original draft (equal). **Guiying Wang:** Methodology (equal); writing – original draft (equal); writing – review and editing (supporting).

## FUNDING INFORMATION

Funding information is not applicable.

## CONFLICT OF INTEREST STATEMENT

The authors declare that they have no competing interests, and all authors should confirm it.

## ETHICS STATEMENT

This study received approval from the Ethics Committee of the Fourth Hospital of Hebei Medical University, and all patients provided informed consent before their inclusion in the study, adhering to ethical guidelines and standards.

## Data Availability

The data used to support the findings of this study are available from the corresponding author upon request.
