# Correction to Comprehensive Analysis of Diagnosis and Treatment in 99 Cases of Abdominal Schwannoma

**DOI:** 10.1002/cam4.71277

**Published:** 2025-09-26

Fan S, Wang H, Sun X, et al. Comprehensive analysis of diagnosis and treatment in 99 cases of abdominal Schwannoma. *Cancer Med*. 2024; 13:e70140. doi:10.1002/cam4.70140

In the originally published version of this article, the article type was incorrectly listed as Review. The correct article type is Research Article.

We apologize for this error.

